# Delayed Hypersensitivity Reaction after Nipple Tattooing: A Novel Case Report

**DOI:** 10.1097/GOX.0000000000002394

**Published:** 2019-09-30

**Authors:** Walter J. Joseph, Eva Roy, Guy M. Stofman

**Affiliations:** From the Department of Plastic Surgery, University of Pittsburgh School of Medicine, Pittsburgh, Pa.

## Abstract

We present a patient who underwent bilateral 3-dimensional (3D) nipple tattooing for nipple areolar reconstruction after implant-based breast reconstruction for breast cancer. Several weeks after nipple tattooing, the patient developed a delayed hypersensitivity reaction around both of her tattooed nipple areolar complexes. This is the first case reported in the literature of a hypersensitivity reaction from 3D nipple tattooing.

## INTRODUCTION

Nipple areolar reconstruction with 3D nipple tattooing is often the final step in the breast reconstruction journey and often considered by women to be the most important.^1^ It has been well described in the literature that patients who undergo nipple areolar reconstruction have a higher degree of postoperative satisfaction than patients who undergo breast reconstruction alone.^2^ Overall, 3D nipple tattooing has been established as a safe procedure that is typically well tolerated with minimal discomfort to the patient due to decreased sensitivity in mastectomy skin flaps.^3^ The 3D tattooing process involves using a variety of pigments to create a 2-dimensional replica of a natural nipple areolar complex (NAC), which gives the appearance of a realistic, 3-dimensional structure.^4^ Here, we present a novel case of a patient who developed a delayed hypersensitivity reaction after 3D nipple tattooing. Although delayed hypersensitivity reactions from tattooing—specifically pertaining to red tattoo ink—have been previously described in the literature, there have been no reported cases of delayed hypersensitivity reactions from nipple tattooing.^5,6^

## CASE

**Fig. 1.**
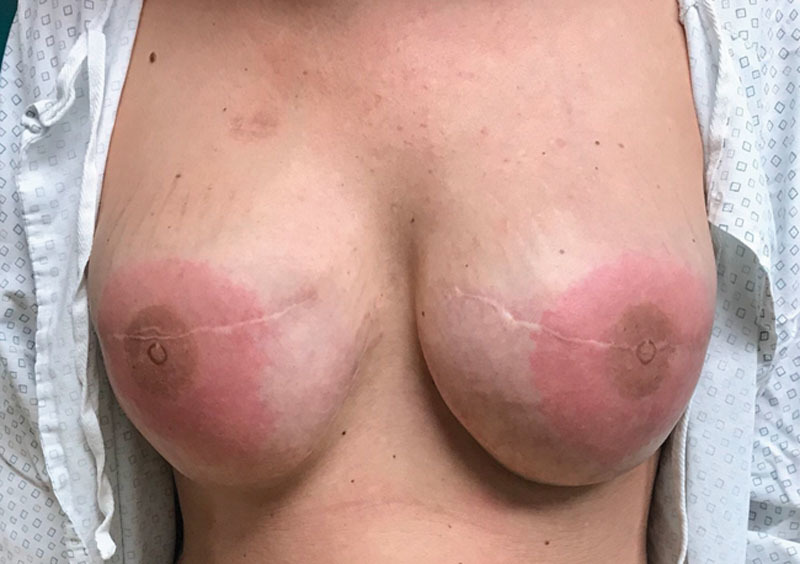
Well-demarcated erythema noted specifically in the distribution of the previously tattooed nipple areolar complexes.

**Fig. 2.**
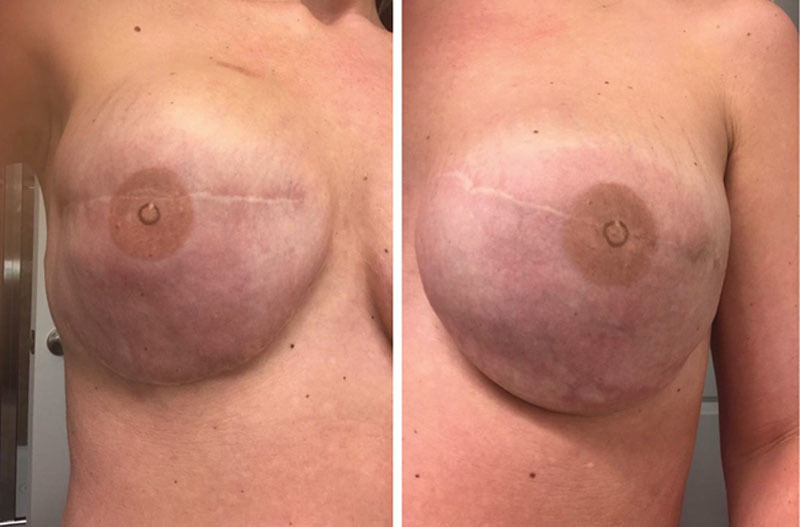
Resolving erythema (as compared to Figure 1) shortly after beginning the Medrol DosePak

**Fig. 3.**
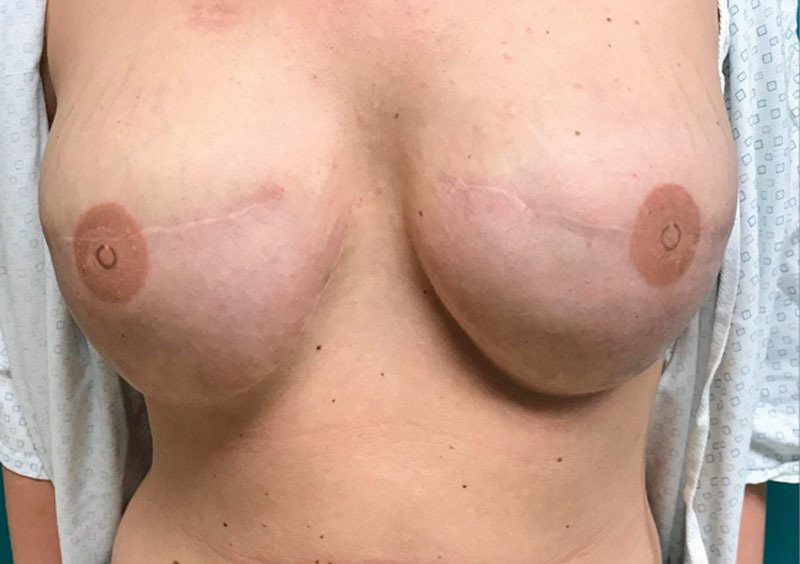
Erythema completely resolved.

A 33-year-old female nonsmoker with a history of breast cancer status post bilateral mastectomies with immediate tissue expander reconstruction presented for bilateral 3D nipple tattooing several months after having her tissue expanders exchanged for silicone implants. The patient did report allergies to penicillin, sulfa, and gluten. At her initial consultation, a “scratch test” was performed using several tattoo inks in the postauricular hairline area to assess for any allergic reaction. The patient did not develop any reaction to the tattoo ink. She returned to clinic 7 weeks after her initial “scratch test” for her definitive tattooing. At that time, NACs were traced on the breasts in appropriate anatomic position and the marked areas were anesthetized with 1% lidocaine with 1:100,000 epinephrine. A Permark coil tattoo device (PMT Corporation, Chanhassen, Minn.) with Electra Pro tattoo ink (Unimax, New York, NY) in 5 colors (brown beige, terra cotta red, chocolate brown, pink beige, cream beige) were used for the 3D tattoo. The procedure was uncomplicated and was tolerated well by the patient. Aquaphor (Beiersdorf Inc., Hamburg, Germany) and Adaptic (Systagenix, San Antonio, Tex.) were applied over the NACs after the procedure. About 7 weeks after nipple tattooing, the patient presented to the clinic with report of an erythematous, pruritic rash around both of her tattooed NACs, which began about 4 days before presentation (Fig. 1). She denied any fevers or other infectious symptoms. Her primary care provider had prescribed topical cortisone, and she had been taking Benadryl (diphenhydramine) with little improvement in her symptoms. She was then prescribed a Medrol DosePak (methylprednisolone, 4 mg). Shortly thereafter, her symptoms began to improve and her rash resolved (Fig. 2). After completion of the Medrol DosePak, the rash completely resolved (Fig. 3).

## DISCUSSION

Nipple areolar reconstruction with 3D tattooing is a common and well-tolerated procedure that improves patient satisfaction and aesthetic outcomes after both autologous and implant-based breast reconstruction.^1,2^ Despite having no reaction to the “scratch test”, our patient developed a delayed hypersensitivity reaction to the nipple tattoo ink approximately 7 weeks after her procedure. The erythema was in a very well-circumscribed distribution surrounding each tattooed NAC and was associated with pruritus. Interestingly, delayed hypersensitivity reactions from tattoos incorporating red ink have been reported in several patients.^5,6^ In a case series by Gaudron et al., several specific red pigments were noted in 6 patients who all experienced a delayed hypersensitivity reaction in the specific distribution of the tattoo ink. Interestingly, 2 of the red pigments described in the aforementioned case series—Cl12475 and Cl73915—were also components of the Electra Pro tattoo inks used on this patient.^7^ Although not previously described in the literature, this case highlights the risk of delayed hypersensitivity reaction in 3D nipple tattooing. Furthermore, we have corroborated prior studies’ reports of a propensity for hypersensitivity reactions from red inks. Although treatments for this reaction are not well studied, most involve topical, intralesional, or oral steroids.^5^ Fortunately, our patient noted full resolution of her symptoms after completing the Medrol DosePak.

## CONCLUSIONS

We have shown for the first time that a delayed hypersensitivity reaction is a possible risk for patients undergoing this adjunctive procedure, specifically when using tattoo inks containing red pigments. Therefore, we urge plastic surgeons as well as medical-grade tattoo artists who are performing 3D nipple tattoos to thoroughly counsel their patients on the possibility of this reaction.
